# The diagnosis dilemma of coexistence of neurosarcoidosis and tuberculosis: case reports and literature review

**DOI:** 10.3389/fmed.2026.1709977

**Published:** 2026-01-30

**Authors:** Qing Sun, Jinsong Jiao, Renbin Wang, Dongyan Wu, Huaping Dai, Shi Shu, Yunchao Su, Dantao Peng, Weihe Zhang, Xiaohui Duan

**Affiliations:** 1 Department of Neurology, China-Japan Friendship Hospital, Beijing, China; 2 Department of Pulmonary and Critical Care Medicine, China-Japan Friendship Hospital, Beijing, China; 3 Department of Pathology, China-Japan Friendship Hospital, Beijing, China

**Keywords:** immunosuppressants, infliximab, neurosarcoidosis, steroids, tuberculosis

## Abstract

Sarcoidosis is a multisystem disease pathologically characterized by non-caseating epithelioid granulomas. Its neurological form, neurosarcoidosis (NS), can affect the central nervous system (CNS) and/or the peripheral nervous system (PNS), posing a significant diagnostic challenge. The diagnostic dilemma is further complicated by the significant clinicopathological overlap between sarcoidosis and tuberculosis (TB). Although rare, sarcoidosis and TB can coexist. Our case series included three NS patients: two had CNS involvement and one had PNS involvement. Notably, two patients had confirmed active tuberculosis infection and one patient had a latent TB infection. This highlights the diagnostic and therapeutic challenges of these coexisting conditions. Fluorodeoxyglucose positron emission computed tomography (FDG PET) helps detect extraneural sarcoidosis locations and find biopsy sites. Regarding the similarity and potential coexistence of TB and sarcoidosis, thorough screening for TB infection is essential before initiating any therapy for sarcoidosis.

## 1 Introduction

Sarcoidosis is a multisystem inflammatory disease pathologically characterized by non-caseating epithelioid granulomas, exhibiting variable clinical manifestations (1, 2). Neurosarcoidosis (NS), the neurological form of sarcoidosis, also shows highly variable clinical manifestations, which can affect the central nervous system (CNS) and/or peripheral nervous system (PNS), with a reported prevalence of 5–10% in all sarcoidosis patients (1, 3). The lack of reliable NS diagnostic biomarkers makes clinical identification particularly challenging (2, 4). Non-caseating epithelioid granulomas are the pathological hallmarks of NS (1). However, granulomatous infections such as tuberculosis (TB) can have similar clinical, radiological, and pathological findings to sarcoidosis (1). Sometimes, it is difficult to differentiate sarcoidosis from TB. Although rare, both conditions can coexist. In this study, we present three cases of NS identified in Asian patients, a population underrepresented in the literature. Of these, two cases demonstrated CNS involvement, and one case demonstrated PNS involvement. Notably, two patients were diagnosed with NS combined with confirmed TB, and one had a latent TB infection. Through a comprehensive literature review, we aim to enhance the differential diagnosis of NS and TB by investigating their diagnostic approaches and treatment strategies.

## 2 Case presentation

### 2.1 Case 1

**Figure 1:**
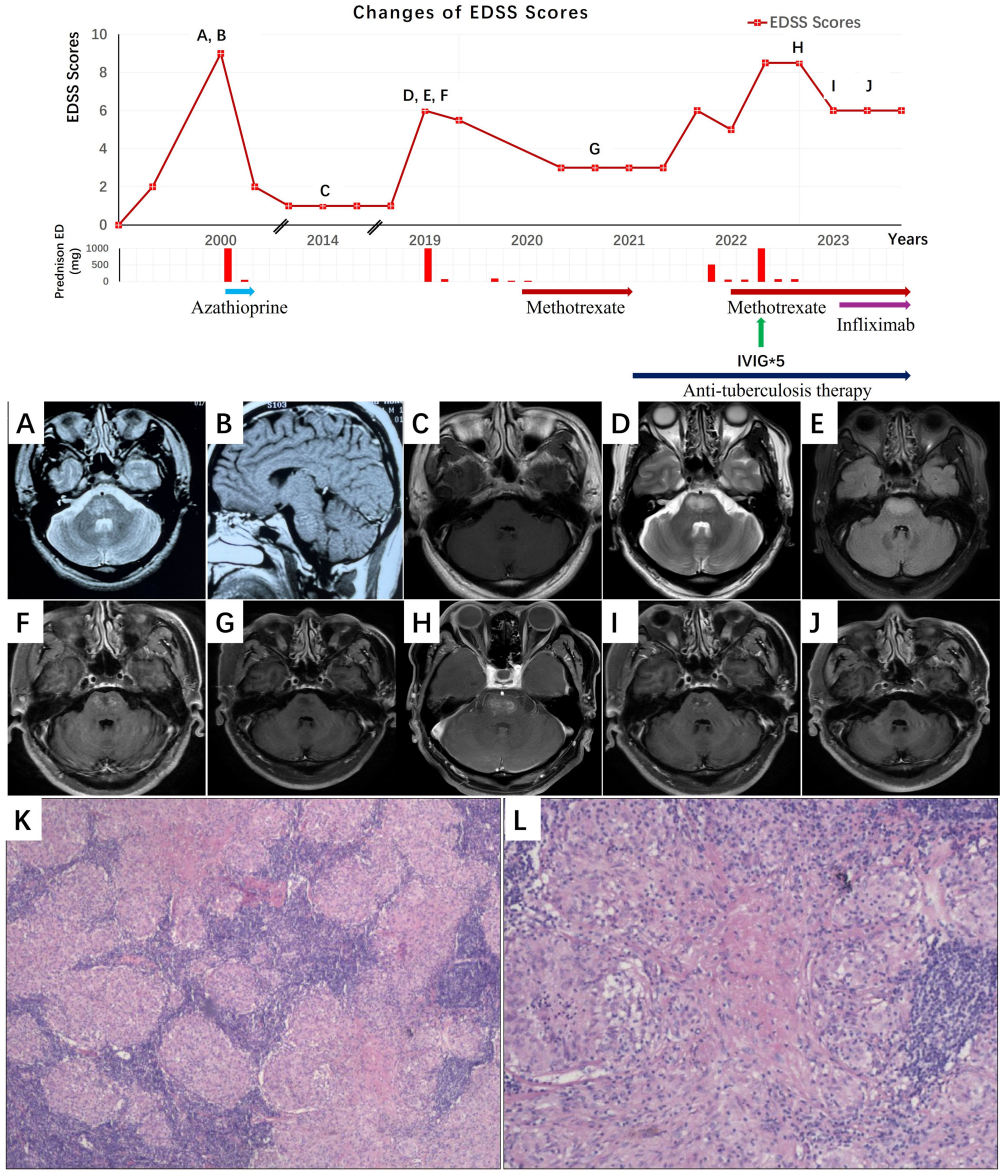
Clinical course, neuroimaging, and pathological findings in Case 1. Serial brain MRI findings are shown in relation to disease progression, relapses, and treatment response. **(A, B)** In the first episode, imaging demonstrates multiple brainstem lesions with patchy enhancement (**A**: axial T2-weighted image; **B**: sagittal gadolinium-enhanced T1-weighted image). **(C)** Resolution of enhancement following the first episode treatment (**C**: axial gadolinium-enhanced T1-weighted image). **(D–F)** In the second episode, recurrent pontine swelling with punctate enhancement was observed (**D**: axial T2-weighted image; **E**: axial T2-FLAIR image; **F**: axial gadolinium-enhanced T1-weighted image). **(G)** Complete resolution of enhancement after the second episode treatment (**G**: axial gadolinium-enhanced T1-weighted image). **(H)** In the third episode, marked pontine swelling with prominent enhancement reappeared (**H**: axial gadolinium-enhanced T1-weighted image). **(I)** Persistent brainstem enhancement despite treatment (I: axial gadolinium-enhanced T1-weighted image). **(J)** Complete resolution of enhancement after infliximab therapy (**J**: axial gadolinium-enhanced T1-weighted image). **(K, L)** Thoracoscopic lymph node biopsy shows a large amount of epithelioid granuloma formation, and multinuclear giant cell reaction (**K**: H&E stain × 100 magnification **L**: H&E stain × 200 magnification). IVIG, intravenous immunoglobulin; prednisone ED, prednisone equivalent dose; EDSS score, Expanded Disability Status Scale; H&E stain, hematoxylin and eosin.

We present the case of a 48-year-old previously healthy man who presented with recurrent limb numbness and weakness for 25 years. The schematic illustration of the disease course is shown in Figure 1. Over these 25 years, the patient experienced three distinct episodes of relapse and remission. In the first episode, which occurred in 2000, the patient was bedridden, and his peak Expanded Disability Status Scale (EDSS) was 9. Physical examination revealed dysphagia and limb weakness, grade 3 on the Medical Research Council (MRC) Scale. Brain magnetic resonance imaging (MRI) showed multiple lesions with patchy enhancement in the bilateral corona radiata, brainstem, and cervical spinal cord (Figures 1A,B). The cerebral spinal fluid (CSF) examination revealed normal pressure, a glucose level of 4.72 mmoL/L, a protein level of 0.19 g/L, and a white blood cell count of 0 cells/μL. He was diagnosed with inflammatory demyelination of the brain stem. The patient was treated with a high dose of intravenous methylprednisolone (IVMP), followed by oral steroids with a tapering dose. Azathioprine was also prescribed. The patient’s symptoms gradually improved, but he had residual limping (EDSS 1). The repeat brain contrast-enhanced MRI was normal (Figure 1C). Although he discontinued all medications several months later, his condition remained stable until 2019.

In 2019, the patient had a second episode, with a peak EDSS score of 6. Physical examination revealed dysphagia, dysarthria, and limb weakness (MRC 4). A brain MRI showed mild swelling of the pons with punctate enhancement (Figures 1D–F). Testing for serum angiotensin converting enzyme (ACE), antinuclear antibody, antineutrophilic cytoplasmic antibody (ANCA), erythrocyte sedimentation rate (ESR), and tumor markers revealed all values to be normal. CSF analysis showed mild pleocytosis (8 cells/μL) with a lymphocyte predominance. Serum and CSF oligoclonal bands were negative. Acid-fast bacteria (AFB) stains and cultures were negative. He was diagnosed with relapsing inflammatory demyelination of the brain stem. The patient started on a high dose of IVMP, followed by oral steroids as maintenance therapy. Despite transient symptom fluctuations during steroid tapering, gradual steroid dose adjustment led to eventual clinical and radiological recovery (EDSS 3) (Figure 1G).

However, during this course, chest computed tomography (CT) revealed multiple enlarged mediastinal lymph nodes, and thoracoscopic lymph node biopsy showed many epithelioid granulomas (Figures 1K,L). The GeneXpert assay, AFB culture, and *Mycobacterium tuberculosis* polymerase chain reaction (MTB PCR) test on the lymph node were all negative for MTB. The biopsy findings were consistent with sarcoidosis, so methotrexate was added. After 6 months of ongoing steroids and methotrexate therapy, a follow-up chest CT showed a reduction in mediastinal lymphadenopathy.

In 2020, the patient developed a pleural effusion and a mass on the chest wall. The GeneXpert testing of empyema was positive for MTB, which is sensitive to rifampin. He was diagnosed with pulmonary TB. He started a regular quadruple anti-TB therapy, consisting of isoniazid, rifampin, pyrazinamide, and ethambutol, and methotrexate was discontinued. The pleural effusion subsequently resolved, and the patient experienced no NS recurrence and retained full functional capacity by 2022.

In 2022, the patient had a third episode, with a peak EDSS score of 8.5. A physical examination revealed ophthalmoplegia, dysphagia, dysarthria, and limb weakness (MRC 2). The CSF analysis showed mild pleocytosis (7 cells/μL) with a lymphocyte predominance. Brain MRI revealed multiple abnormal signals in the pons (Figure 1H). Chest CT remained stable, and repeat sputum induction (NGS Xpert, AFB) was negative. The T-SPOT. TB was positive. Given the recurrence of NS and the history of active TB, the patient received two courses of IVMP and one course of intravenous immunoglobulin (IVIG) therapy, combined with anti-TB therapy. Methotrexate was reintroduced. His symptoms partially improved (EDSS 6), and the brain MRI indicated that the pons enhancement had disappeared. However, he remained partially dependent, requiring bilateral crutches for ambulation. In 2023, a gadolinium contrast-enhanced brain MRI showed persistent enhancement of the pons lesions without worsening of clinical symptoms (Figure 1I). Infliximab was initiated while continuing methotrexate. Six months later, the enhancement of the pons lesions had disappeared (Figure 1J).

### 2.2 Case 2

We present the second case of a 54-year-old previously healthy woman who was admitted with complaints of progressive lymph node enlargement for 10 years and of headache and diplopia for 3 years. Ten years before admission, the patient noticed progressive enlargement of cervical lymph nodes without seeking formal medical evaluation or treatment. Four years before admission, she developed a persistent cough and chest pain. Chest and abdomen CT revealed enlarged mediastinal and retroperitoneal lymph nodes. A biopsy of cervical lymph nodes in a local hospital suggested chronic granulomatous inflammation with caseous necrosis, T-SPOT. TB and AFB stains were positive. Based on these findings, she was diagnosed with TB and received standard quadruple anti-TB therapy with isoniazid, ethambutol, rifampicin, and pyrazinamide. However, she had no clinical or radiological response to anti-TB therapy.

**Figure 2:**
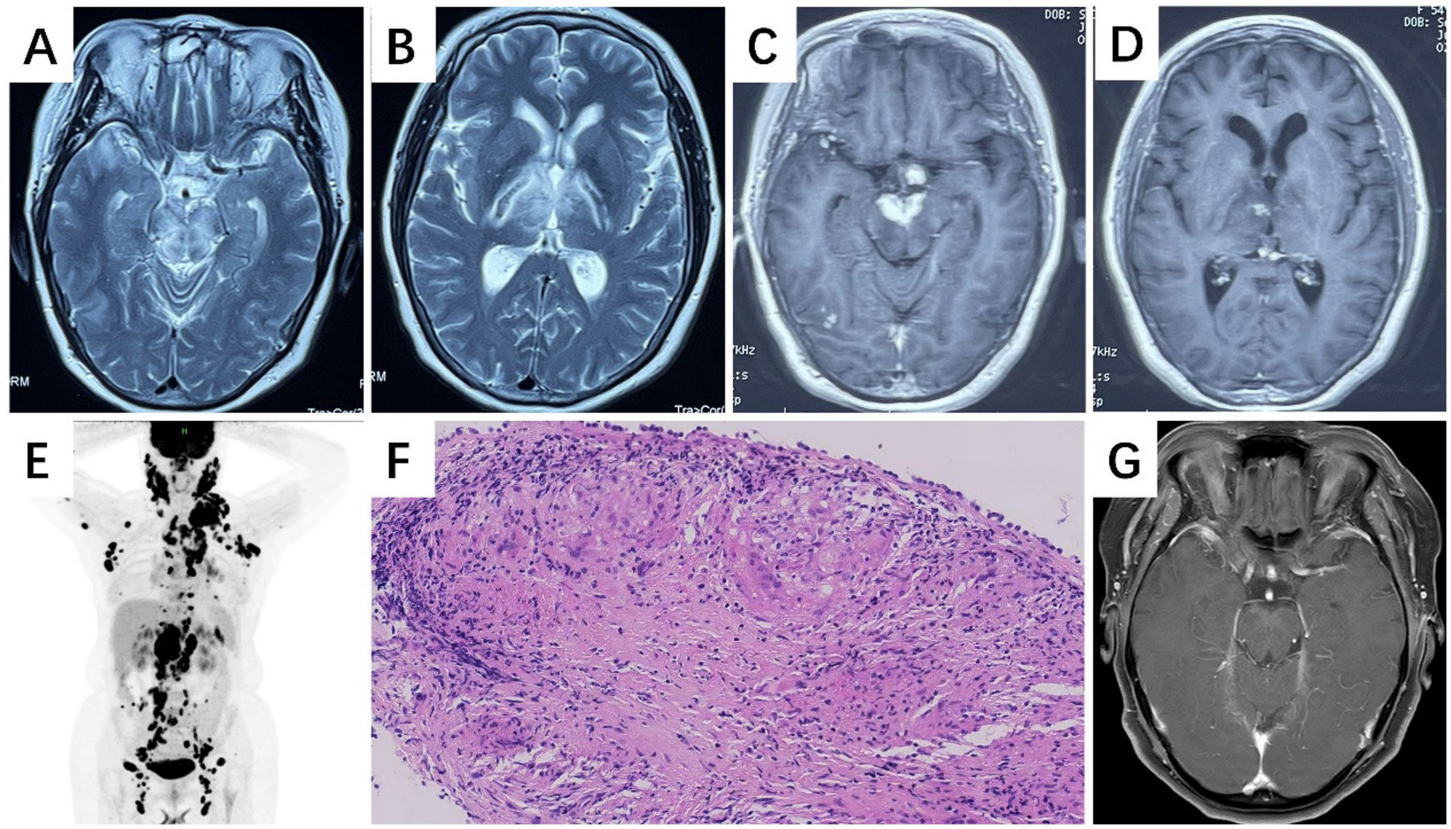
Neuroimaging and pathological findings in Case 2. **(A–D)** Brain MRI at initial presentation demonstrates lesions with enhancement in the pons, midbrain, and bilateral thalami (**A, B**: axial T2-weighted images; **C, D**: axial gadolinium-enhanced T1-weighted images). **(E)** FDG PET reveals multiple hypermetabolic nodules. **(F)** Mediastinal lymph node biopsy shows non-caseating epithelioid granulomas (H&E stain, ×20 magnification), consistent with sarcoid-like granulomatous inflammation. **(G)** Follow-up brain MRI after treatment demonstrates a marked reduction in lesion enhancement, indicating therapeutic response. FDG PET, fluorodeoxyglucose positron emission computed tomography; H&E stain, hematoxylin and eosin.

Three years before admission, the patient presented with a headache, blurred vision in the right eye, and diplopia. Physical examinations revealed right eye ptosis and external ophthalmoplegia. The brain MRI showed abnormal signals with enhancement in the hypothalamus, pontine, midbrain, and bilateral thalamus (Figures 2A–D). CSF analysis revealed normal pressure, a glucose level of 2.63 mmoL/L, a protein level of 0.44 g/L, and a white blood cell count of 1 cell/μL. Fluorodeoxyglucose positron emission computed tomography (FDG PET) examination showed multiple hypermetabolic nodules (Figure 2E). Repeat biopsies (via thoracoscopy and cervical node excision) showed epithelioid granulomatous inflammation but without caseous necrosis (Figure 2F). MTB PCR test, GeneXpert assay, and AFB culture from these new specimens were negative. Given the progression of neurological symptoms despite an initial diagnosis of TB and the lack of response to anti-TB therapy—along with the new findings of non-caseating granulomas and negative microbiological tests—a separate, treatment-refractory granulomatous process was suspected. The patient was diagnosed with neurosarcoidosis (NS), with a history of presumed prior TB infection. She started on prednisone and methotrexate, which led to the shrinkage of the lung lymph nodes. However, her headache and blurred vision did not improve. She then started on IVMP, followed by a tapering course of oral steroids, and mycophenolate mofetil was also added. Subsequently, her headache and ophthalmoplegia improved, and follow-up brain MRI showed a significant reduction in enhancement (Figure 2G).

### 2.3 Case 3

**Figure 3:**
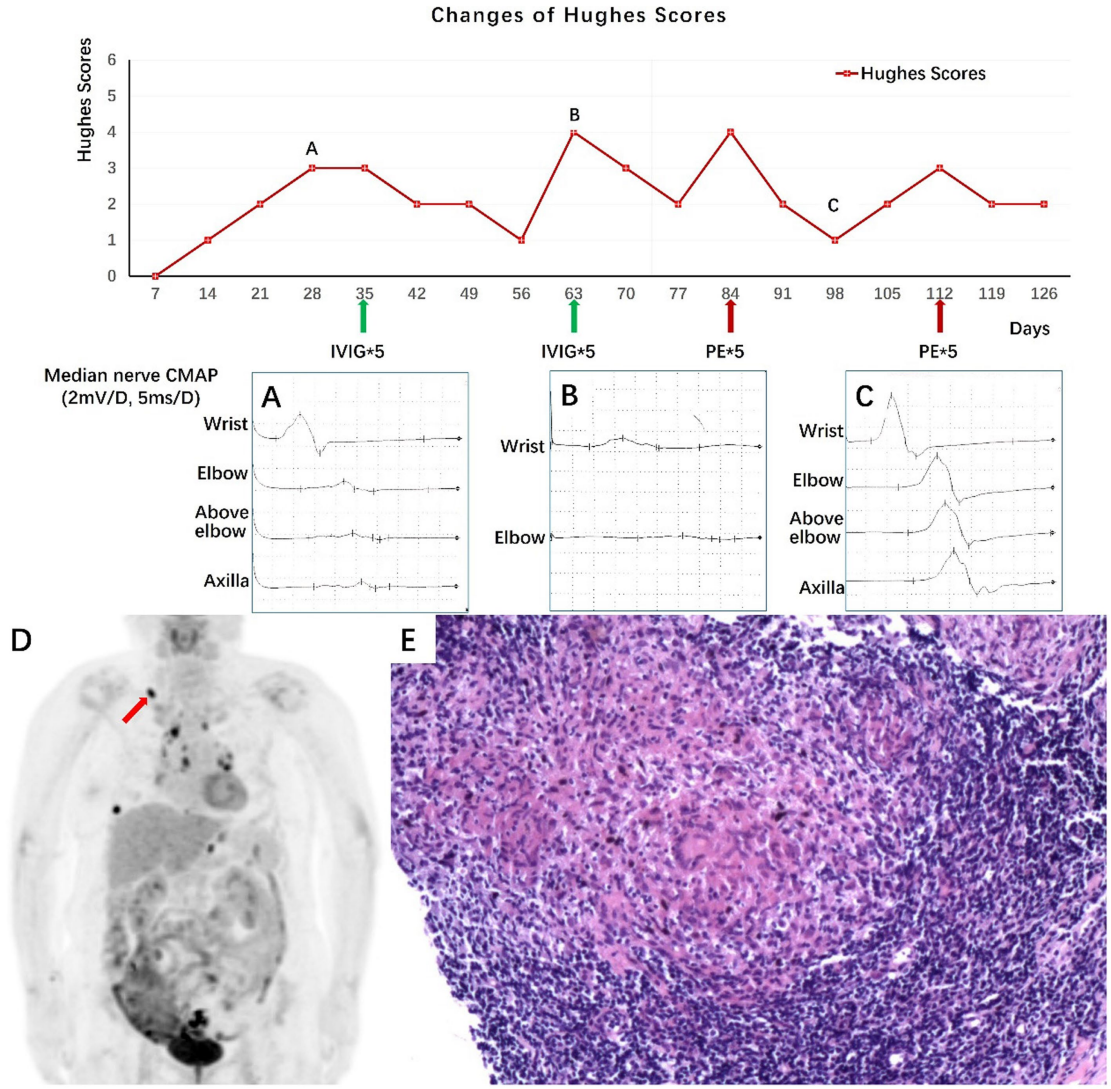
Schematic illustration of the disease course of Case 3 shows the temporal sequence of Hughes scores, median nerve motor conduction, and treatment. **(A)** Median nerve motor conduction block, consistent with demyelinating changes. **(B)** Worsening in median nerve CMAP amplitude. **(C)** Significant improvement in median nerve CMAP amplitude. **(D)** FDG PET shows increased glucose metabolism in multiple lymph nodes at the bottom of the stomach, right supraclavicular fossa, and mediastinum. **(E)** Right supraclavicular fossa lymph node biopsy shows epithelioid granulomatous inflammation without caseous necrosis (H&E stain × 200 magnification). IVIG, intravenous immunoglobulin; PE, plasma exchange; CMAP, compound muscle action potential; FDG PET, fluorodeoxyglucose positron emission computed tomography.

We present a third case of a 66-year-old woman with a history of hypertension, diabetes mellitus, and bronchiectasis who was admitted for recurrent limb numbness and weakness for 4 months. A schematic illustration of the disease course of Case 3 is shown in Figure 3. The patient experienced four distinct relapse-remission episodes in 4 months. Four months before admission, the patient had the first attack of progressive limb numbness and weakness, peaking at Hughes Functional Grading Scale of 3. Physical examinations revealed limb weakness (MRC 3) and areflexia. Electromyography showed peripheral sensory and motor nerve demyelination (Figure 3A). CSF analysis revealed normal pressure, a glucose level of 4.34 mmoL/L, a protein level of 0.28 g/L, and a white blood cell count of 0 cells/μL. She was diagnosed with “Guillain-Barré Syndrome” and was treated with IVIG, achieving significant improvement (Hughes 1, MRC 5).

Two months before admission, the patient had a second attack of limb weakness (Hughes 4, MRC 2). Repeat CSF analysis revealed normal pressure, a glucose level of 3.78 mmoL/L, a protein level of 0.47 g/L, and a white blood cell count of 4 cells/μL. A second IVIG course led to significant improvement (Hughes 2, MRC 4).

One month before admission, the patient had the third attack (Hughes 4, MRC 2). Antinuclear antibody profile and tumor markers were normal. Serum immunofixation electrophoresis did not reveal M protein. ESR was 38 mm/h. T-SPOT. TB was strongly positive (antigen A: 180 SFCs/2.5 × 10^5^ PBMCs; antigen B: 203 SFCs/2.5 × 10^5^ PBMCs; normal range < 6 SFCs), without other evidence of active TB. Chest CT showed bronchiectasis and pulmonary infection. Electromyography confirmed persistent demyelinating changes (Figure 3B). Given the relapsing–remitting course, the diagnosis was revised to chronic inflammatory demyelinating polyneuropathy (CIDP). Considering the high risk of exacerbating her bronchiectasis and reactivating latent tuberculosis, steroid and conventional immunosuppressant therapy were withheld. Instead, she underwent plasma exchange (PE), resulting in marked clinical and electrophysiological improvement (Hughes 1, MRC 5) (Figure 3C).

One week after PE treatment, the patient experienced the fourth episode (Hughes 3, MRC 4). An FDG-PET scan revealed hypermetabolic lymph nodes at the bottom of the stomach, right supraclavicular fossa, and mediastinum (Figure 3D). Gastroscopy revealed chronic gastritis. A biopsy of the right supraclavicular fossa lymph node showed non-caseating epithelioid granulomatous inflammation, which was consistent with sarcoidosis (Figure 3E). This raised the possibility that her immune-mediated neuropathy could be a manifestation of NS or occur concurrently with systemic sarcoidosis. A sural nerve biopsy showed epineural edema without vasculitis or significant inflammation; however, non-caseating granulomas were not found. Given the recurrent and treatment-refractory nature of the neuropathy in the setting of newly diagnosed systemic sarcoidosis, immunosuppressant cyclophosphamide was initiated despite the infection risks. Unfortunately, the patient died of a severe infection 1 year later.

## 3 Discussion

Sarcoidosis is a multisystem inflammatory disease of unclear etiology (5). The disorder can affect any organ in the body, and it can have a wide range of clinical manifestations (5). NS may affect any part of the central and peripheral nervous system (6). The diagnosis of sarcoidosis is hampered by a lack of reliable diagnostic biomarkers (2). In 2018, the Neurosarcoidosis Consortium Consensus Group proposed definitions of possible, probable, and definite NS (7). According to the diagnostic criteria, definite NS is defined as a nervous system pathology consistent with NS. However, achieving a definite diagnosis of NS is challenging due to the relative inaccessibility of tissue for biopsy (4). The risk of performing biopsies on a vulnerable structure, such as the brainstem, needs to be weighed against the potential consequences of an incorrect diagnosis (8). In such cases, it is crucial to conduct a thorough investigation into systemic extraneural sarcoidosis using CT and FDG PET scans (6). FDG PET is the most effective for detecting sarcoidosis inflammatory activity and identifying sites for diagnostic biopsies (5). Systemic extraneural proof of sarcoidosis, coupled with the appropriate neurologic syndrome, is sufficient to establish a diagnosis of probable NS (9). When superficial lesions, such as a skin nodule or enlarged peripheral lymph node, are unavailable, an intrathoracic biopsy is commonly used (2). Bronchoscopy, with transbronchial or bronchial biopsies, is positive for the presence of non-caseating granulomas in 50–85% of sarcoidosis patients (2). In our study, all three cases were diagnosed through lymph node biopsy, with the biopsy sites being identified by CT and/or FDG PET scans: two via thoracoscopic lymph node biopsy and one via peripheral lymph node biopsy. The pathology of the three patients all revealed epithelioid granulomatous inflammation without caseous necrosis, consistent with the definition of probable NS. In our case reports, two cases (Cases 1 and 2) involved CNS, and one case (Case 3) involved PNS.

The clinical manifestations of CNS NS include cranial neuropathy, mass lesions, meningitis, encephalitis, myelitis, and hypothalamic–pituitary involvement (2). MRI findings in CNS NS range from basal involvement (meningeal, hypothalamic infundibular, and cranial nerve) and hydrocephalus to parenchymal and dural mass lesions (8). The enhancement pattern can be diffuse, nodular, or spread along perivascular spaces (8). Basal or widespread leptomeningeal involvement is the typical characteristic of NS (8). The two CNS cases (Cases 1 and 2) presented with different clinical manifestations and imaging characteristics: Case 1 presented as recurrent pontine inflammation, a rare imaging manifestation of NS, whereas Case 2 presented with basal or diffuse leptomeningeal involvement, representing its more typical imaging presentation.

Peripheral nervous system sarcoidosis is rare, accounting for approximately 14% of all neurological involvements (10). Its clinical presentation is diverse, encompassing polyneuropathy, multiple mononeuropathies, radiculoneuropathy, small fiber neuropathy, and autonomic neuropathy (10). The typical pathological characteristic of sarcoid neuropathy is granulomatous inflammation in the vascular walls of the sural nerve epineurium (11). Within the spectrum of sarcoid neuropathy, CIDP is a rare manifestation that can closely mimic idiopathic CIDP, particularly in patients with no prior diagnosis of sarcoidosis (10). Case 3 presented with recurrent CIDP, and a lymph node biopsy confirmed sarcoidosis, meeting the criteria for probable NS. However, the sural nerve biopsy in this case did not reveal non-caseating granulomas. We propose two potential explanations for this finding: First, given the sampling limitations of sural nerve biopsy, the granulomatous lesions may have been missed. Alternatively, this case may represent a concomitant occurrence of sarcoidosis and CIDP. Sarcoidosis could serve as a precipitating factor for autoimmune demyelinating neuropathy, even in the absence of direct granulomatous infiltration of peripheral nerves. Previous studies have suggested that humoral factors, such as matrix metalloproteinases produced during granulomatous inflammation, may contribute to myelin degradation (11).

Sarcoid granulomas are typically non-caseating, although they may exhibit small to moderate amounts of fibrinoid necrosis (9, 12). However, granulomatous inflammation is also associated with infectious diseases such as TB, leprosy, and schistosomiasis (13). TB is pathologically characterized by granulomas with caseous necrosis (14). However, some TB granulomas can present in a non‑caseating form, and even the pathological distinction between caseating and non‑caseating granulomas is not always absolute — creating a diagnostic dilemma in differentiating sarcoidosis from tuberculosis (14). Sarcoid granulomatosis and TB are sometimes difficult to differentiate, even on pathological grounds, in the absence of cultural microbiological confirmation. Rigorous screening of infectious granulomatous disorders is essential to avoid overlooking an alternative infectious etiology (9).

Both Cases 1 and 2 were diagnosed with the occurrence of sarcoidosis and TB, posing significant diagnostic and therapeutic challenges. Both steroids and immunosuppressants, cornerstones of sarcoidosis treatment, can exacerbate TB, facilitate its spread, and complicate the management of sarcoidosis. In Case 1, lymph node biopsy findings were consistent with NS. However, due to recurrent relapses, the patient received repeated courses of glucocorticoids and methotrexate therapy without prior tuberculosis (TB) screening or prophylactic treatment, and finally, this ultimately led to the development of active pulmonary TB, creating a critical therapeutic dilemma. After withdrawing immunosuppressants and initiating anti-TB therapy, TB was controlled. Notably, sarcoidosis remained in remission during this period. When brainstem lesions later recurred, a lumbar puncture with AFB PCR and MTB culture was performed to exclude CNS TB. No evidence of CNS TB was found, and the brainstem lesions were attributed to NS. Steroids and immunosuppressants were subsequently reintroduced, and anti-TB therapy was maintained as prophylaxis for high-risk TB reactivation. Case 1 served as a reminder of the risk of TB reactivation in immunocompromised patients and highlighted a potentially preventable complication that could have been avoided with proper TB screening and management before and during immunosuppressive treatment. In Case 2, the initial symptom was lymph node enlargement, leading to a diagnosis of microbiologically confirmed TB and subsequent anti-TB treatment. One year later, the patient developed CNS symptoms. Basilar meningeal involvement can be indicative of either tuberculous meningitis or NS, posing a significant diagnostic challenge. A lymph node biopsy showed epithelioid granulomatous inflammation without caseous necrosis. Extensive laboratory investigations, including CSF analysis, yielded limited evidence for active TB. Consequently, the patient was diagnosed with NS with a history of presumed prior TB infection. After treatment with steroids and immunosuppressants, the patient’s clinical symptoms were in remission. Case 3 had a positive T-SPOT. TB test, without evidence of active TB, suggesting prior or latent TB infection. Given the concurrent presence of bronchiectasis, pulmonary infection, and a positive T-SPOT. TB test, steroids and immunosuppressive therapy were initially withheld due to the high risk of exacerbating the infection. However, after multiple disease recurrences, cyclophosphamide was ultimately initiated. Tragically, the patient subsequently succumbed to a severe pulmonary infection.

Although rare, the co-occurrence of sarcoidosis and TB is well-documented, as summarized in Table 1. Sarcoidosis was pathologically confirmed across multiple organ systems: pulmonary (four cases), cutaneous (three cases), mediastinal lymph nodes (two cases), inguinal lymph node (one case), combined pulmonary and bone marrow involvement (one case), and combined cutaneous and pulmonary involvement (one case). Microbiological evaluation confirmed active TB infection in 11 patients through various methods: one case demonstrated concurrent MTB positivity in both bronchoalveolar lavage (BAL) fluid and lymph node AFB smear, 6 cases showed MTB detection in BAL via PCR, GeneXpert, or culture, 2 cases showed AFB smear positivity (in lymph node biopsy and sputum, respectively), and 2 cases showed MTB culture positivity (in pleural specimens and bronchial aspirates, respectively). The remaining case (No. 3) was diagnosed with latent TB infection. Following combined anti-TB therapy and steroid treatment, 11 patients achieved clinical and radiological resolution, while 1 patient died of sepsis. All cases illustrate the complex spectrum of interactions between these diseases, ranging from latent TB infection in the setting of NS to simultaneous active disease, and underscore the critical importance of meticulous investigation and individualized management strategies. Furthermore, emerging evidence indicates bidirectional links between TB and sarcoidosis: TB patients have an 8.09-fold higher risk of developing sarcoidosis than non-TB patients, and sarcoidosis patients have a 1.85-fold higher risk of TB than non-sarcoidosis patients (12). Additionally, MTB has been detected in 26.4–50% of sarcoidosis patients (15, 16). The similar pathological appearance of the granulomas and the documented coexistence of TB and sarcoidosis suggest possible connections between the two entities (16). Although the precise etiology of sarcoidosis remains unclear, current evidence implicates genetic susceptibility, environmental factors, and transmissible infectious agents as potential contributors (12, 13). Several studies have identified a prior mycobacterial infection in a subset of sarcoidosis patients, and growing evidence suggests that mycobacteria may act as a triggering antigen, prompting an immune response that could eventually lead to sarcoidosis, particularly in genetically predisposed individuals (2, 13–15). Sarcoidosis is a pathobiological outcome of a mycobacterial infection, characterized by a hyperimmune antimycobacterial response that confers persistent immunity and prevents reactivation (2).

**Table 1:** Published cases of concurrent sarcoidosis and tuberculosis in patients.

No.	Author, year	Age, Gender	Tuberculosis findings	Sarcoidosis findings	Prognosis
1	N. Francis 2023 (14)	12, F	Positive AFB smear and PCR in cervical lymph node biopsy; Positive BAL PCR MTB, and GeneXpert	Skin granulomas, no improvement of symptoms with anti-TB medications. Good response to prednisolone	Resolution
2	*C. carbonelli* 2017 (17)	45, M	Positive MTB cultures on pleural specimens	Skin necrotizing granuloma. Good response to prednisolone	Resolution
3	T. C. Hsiung 2014 (18)	35, M	Positive tuberculin skin test with purified protein derivative	Lung necrotizing sarcoid granulomatosis, good response to prednisolone	Resolution
4	S. K. Mandal 2014 (19)	38, F	Positive BAL MTB culture	Bone marrow and lung biopsy granulomas, response to steroids	Resolution
5	G. Dai 2024 (20)	43, F	Positive BAL MTB	Bronchus biopsy non-necrotic epithelioid granulomas	Resolution
6	G. Dai 2024 (20)	50, F	Positive BAL Gene Xpert	Inguinal lymph node non-necrotic epithelioid granulomas	Resolution
7	K. A. Yusuf 2023 (21)	57, M	Positive BAL PCR MTB	Skin granulomas	Resolution
8	H. Kaur 2021 (22)	38, M	Positive MTB culture of bronchial aspiration	Bronchial wall biopsy: non-caseating, chronic granulomas	Resolution
9	K. Mise 2010 (23)	43, F	Positive BAL MTB culture	Skin and lung sarcoidosis	Resolution
10	A. Tufan 2004 (24)	56, F	AFB detected in cervical lymph node	Mediastinal lymph node granuloma consistent with sarcoidosis	Died due to sepsis
11	H. S. Cho 2021 (25)	46, M	Positive sputum AFB smear	Mediastinal lymph node biopsy non-caseating granulomas	Resolution
12	K. C. Hansen 2019 (26)	54, M	MTB detected in BAL fluid and testicle	Lung non-necrotic granuloma	Resolution

F, female; M, male; AFB, acid-fast bacilli; PCR, polymerase chain reaction; BAL, bronchoalveolar lavage; MTB, *Mycobacterium tuberculosis*.

The treatment of NS remains a clinical challenge due to its heterogeneous manifestations and variable severity (2). Steroids are generally considered first-line therapy for NS and tend to work rapidly in most patients (1). However, their long-term use is limited by dose-dependent adverse effects that accumulate over time (5). Furthermore, a significant proportion of NS patients may be refractory to corticosteroids or experience relapses during dose tapering (1). Consequently, several steroid-sparing agents have been employed in the treatment of NS, including azathioprine, methotrexate, mycophenolate mofetil, hydroxychloroquine, cyclophosphamide, and TNF inhibitors. Given the heterogeneous nature of NS, treatment should be individualized (1). Therapeutic regimens differed across the three cases: Case 1 initially received azathioprine followed by methotrexate, and Case 2 responded well to mycophenolate mofetil, while Case 3 was managed with cyclophosphamide. Despite immunotherapy, Case 1 demonstrated persistent post-contrast enhancement on imaging, indicating ongoing active inflammation that may contribute to recurrent disease episodes and progressive tissue injury. Treatment for refractory NS in this case was ultimately escalated to TNF inhibitors (1). Infliximab, a TNF-alpha antagonist, effectively suppresses granuloma formation in sarcoidosis and has been reported to achieve clinical stability or improvement in 87–96% cases (1). During the infliximab treatment course, methotrexate continued, and steroids were weaned off due to their risk of long-term side effects. Immunogenicity, characterized by the development of neutralizing antibodies against infliximab, occurs in a minority of patients and is associated with reduced drug efficacy related to increased infliximab clearance or neutralization (1). In clinical practice, patients with NS continue a lower-dose cytotoxic immunosuppressant, such as methotrexate or azathioprine, alongside infliximab to attenuate the formation of infliximab antibodies in addition to its potential synergistic immunosuppressive benefits (1). However, emerging data indicate that TNF inhibitor monotherapy — that is, use without concurrent use of other immunosuppressants — may also yield favorable outcomes in NS (1).

A key limitation of this study is the small sample size. NS is uncommon, and its co-occurrence with TB is even rarer. Furthermore, sarcoidosis is less prevalent in Asian populations, particularly among Chinese individuals, further limiting the sample size. Future research should focus on expanding the sample size through multicenter collaborations, especially in Asian and Chinese populations, to yield more definitive conclusions.

## 4 Conclusion

The clinical manifestations of NS are heterogeneous and can involve the CNS and/or PNS. The diagnosis of NS is challenging due to the absence of specific biomarkers and the limited accessibility of affected tissue for biopsy. Typically, diagnosis requires evidence of non-caseating granulomas in peripheral tissues. FDG PET can be used for the detection of extraneural sarcoidosis localizations and the identification of extraneural biopsy sites. The clinical, radiological, and pathological similarities between NS and TB complicate diagnosis and management. Although the coexistence of TB and sarcoidosis is rare, it can occur, making rigorous TB screening essential to avoid missing an alternative infectious etiology. Infliximab may provide an effective treatment option for refractory NS.

## Data Availability

The original contributions presented in the study are included in the article/supplementary material, further inquiries can be directed to the corresponding author/s.
